# P04.66 Comparing attitudes preceding and succeeding complementary and alternative medicine and integrative medicine undergraduate courses

**DOI:** 10.1186/1472-6882-12-S1-P336

**Published:** 2012-06-12

**Authors:** A Reeves, N Hunyh, M Liu

**Affiliations:** 1 University of California, Irvine, Irvine, USA

## Purpose

The objective of this study was to determine the changes of attitudes, motivations, and perceptions of Complementary and Alternative Medicine (CAM) and Integrative Medicine (IM) before and after taking CAM/IM related courses at University of California, Irvine. It was hypothesized that with more exposure and education in CAM/IM throughout the course student’s perceptions, attitudes and willingness to learn more about CAM/IM would increase.

## Methods

This was a longitudinal, cross sectional questionnaire-based study conducted on the undergraduates enrolled in CAM/IM related classes offered at UCI. This portion of the study was conducted on the courses “The Biology of Integrative Medicine” in the School of Biological Sciences, and “Global Health and Nutrition” in the College of Health Sciences. An IRB approved survey, additionally supported by the Undergraduate Research Opportunities Program (UROP) was administered to students before and after they had taken the course. Descriptive and comparative analysis was done using SPSS 16.

## Results

Sixty-one percent of 326 respondents in the classes had used CAM/IM. The most commonly used forms of CAM were vitamins/minerals (30%), chiropractic (28%), and body movement (22%). Before the class, most students indicated interest in taking more CAM/IM classes (83%) in the following ways: fulfilling the requirement for graduation (32%), fulfilling a general education requirement (27%), or as a CAM/IM minor (13%). After the classes, 74% would have liked to take more classes on CAM/IM in the following forms: a graduation requirement (27%), for units only (9%), or as a major (9%). Students rating of CAM/IM effectiveness did not change throughout the course.

## Conclusion

In general, it was found that the two classes had a positive effect on students’ use, perception, and desire for further education in CAM/IM. Additionally, the classes seemingly had little effect on the student’s already strong willingness to proceed in taking more classes on CAM/IM.

